# Pilot *Anopheles gambiae *full-length cDNA study: sequencing and initial characterization of 35,575 clones

**DOI:** 10.1186/gb-2005-6-4-r39

**Published:** 2005-03-15

**Authors:** Shawn M Gomez, Karin Eiglmeier, Beatrice Segurens, Pierre Dehoux, Arnaud Couloux, Claude Scarpelli, Patrick Wincker, Jean Weissenbach, Paul T Brey, Charles W Roth

**Affiliations:** 1 Unité de Biochimie et Biologie Moléculaire des Insectes and CNRS FRE 2849, Institut Pasteur, 75724 Paris Cedex 15, France; 2 Genoscope/Centre National de Séquençage and CNRS UMR 8030, 91057 Evry Cedex, France; 3 Plate-forme Intégration et Analyse Génomiques, Institut Pasteur, 75724 Paris Cedex 15, France

## Abstract

We describe the preliminary analysis of over 35,000 clones from a full-length enriched cDNA library from the malaria mosquito vector *Anopheles gambiae*. The clones define nearly 3,700 genes, of which around 2,600 significantly improve current gene definitions. An additional 17% of the genes were not previously annotated, suggesting that an equal percentage may be missing from the current *Anopheles *genome annotation.

## Abstract 2

A preliminary analysis of over 35,000 clones from a full-length enriched cDNA library from the malaria mosquito vector *Anopheles gambiae* identifies nearly 3,700 genes, including a large number of genes that had not been annotated previously.

## Background

Malaria is currently considered to be the most important tropical disease, afflicting 300-500 million people, and killing over 1 million annually [1]. It is caused by infection of the human host with a single-celled parasite belonging to the genus *Plasmodium *and relies on female mosquitoes of the genus *Anopheles *for its transmission. The recent whole-genome sequencing of *Anopheles gambiae*, the primary vector in sub-Saharan Africa of *Plasmodium falciparum *- the agent of the most common and deadly type of malaria - now provides researchers with a vast set of data with which to better understand this insect vector and to develop possible solutions to malaria [2].

Annotation of the *A. gambiae *genome by defining genes and other genomic features is the first step in moving from the realm of simply a genome sequence to one of understanding gene function. Extremely important to this effort is the accumulation of high-quality sequence data capable of refining the structural features of known genes and revealing previously unknown genes. Unfortunately, before the completion of the genome sequence, very few *Anopheles *genes were well characterized experimentally, with exceptions primarily being genes involved either in olfaction or in host-parasite interactions (for example, innate immunity genes).

While the amount and quality of publicly available sequence data is improving, a second complete *Anopheles *gene build in October of 2003 by Ensembl was able to utilize only 40,000 expressed sequence tag (EST) sequences in the EST gene build, leaving gene predictions heavily reliant on finding regions homologous with *Drosophila*, an organism that diverged from *Anopheles *more than 250 million years ago [3-5]. A recent preliminary analysis of the *Anopheles *genome annotation suggests that this lack of sequence data, combined with potential assembly problems and the absence of a closely related organism to use in homology comparisons, is proving a significant challenge for current attempts at genome annotation [6]. Like other groups [7,8], we have initiated a program to increase the total amount of experimental sequence data and improve current *Anopheles *gene models. Unlike EST data, full-length cDNA libraries are biased toward complete copies of mRNA transcripts and thus provide significantly more information, including intron-exon structure as well as the first and last exons (often the most difficult to identify *in silico *[9]), alternative splicing, the correct start codon(s), and the full protein-coding sequence. Additionally, full-length transcripts can be used in the optimization of gene-expression studies and can be used directly as templates for protein synthesis.

Here we report the sequencing and preliminary analysis of 67,044 reads from a full-length enriched cDNA library derived from whole-body adult female *A. gambiae *mosquitoes. These sequences were initially clustered with each other and then aligned to the *Anopheles *genome sequence, and correspond to approximately 3,700 genes. Nearly 650 of these genes appear to be novel because they neither overlap nor simply extend previous Ensembl gene models. In addition, clusters that matched previous gene definitions improved those definitions in 85% of cases. These results demonstrate both the usefulness of full-length cDNAs in genome annotation, as well as the degree to which further annotation of the *Anopheles *genome is needed.

All sequences from this project were submitted to GenBank under the accession numbers BX005485-BX072528 and the physical clones are being submitted to the Malaria Research and Reference Center (MR4) [10].

## Results and discussion

**Figure 1:**
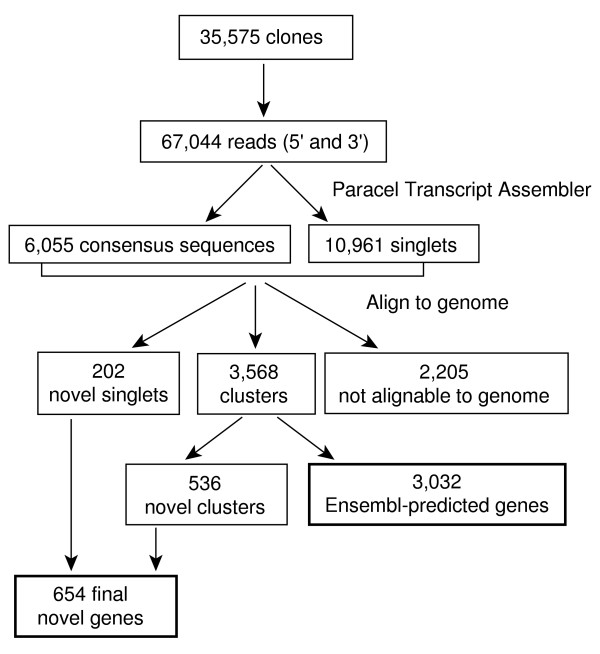
Flow chart of sequence processing and categorization.

We constructed a non-normalized library of enriched full-length cDNAs with RNA extracted from the complete body of adult female mosquitoes (see Materials and methods). Sequencing of clones was carried out from both the 5' and 3' ends of the cDNA insert. After sequencing, sequence reads were cleaned, clustered and assembled into consensus sequences using the Paracel Transcript Assembler package. Output from this process results in the creation of either a single consensus sequence or multiple consensus sequences (because of alternative splicing, for example) for each cluster of overlapping cDNAs. Individual reads that could not be initially clustered with any other sequence are referred to as singlets. Together, consensus and singlet sequences were aligned to the genome, and for each strand, overlapping cDNA sequences were grouped into a single cluster representing a putative gene. This process generates three major end products: clusters and singlets that overlap previously predicted gene models; novel clusters or singlets that do not overlap a gene model; and consensus sequences/singlets that do not align anywhere within the genome. A graphical summary of the analysis is shown in Figure 1. Note that we use the Ensembl 'unknown' chromosome as part of this analysis (designated asUNKN). This artificial chromosome contains arbitrarily ordered concatenated scaffolds that currently have not been assigned to a particular chromosomal location.

### Comparison with previously predicted genes

To discern clusters representing known genes from those that would potentially be considered novel, we compared the coordinates on the genome of our cDNA clusters to those of Ensembl transcript models. Specifically, we used transcript model data taken from Ensembl gene build version 16.2.1, which did not have our cDNAs available for the creation of its 14,653 gene models. In this analysis, clusters were categorized as previously predicted if any overlap occurs, by even a single base, between a cluster and an Ensembl transcript. If no overlap occurs, genes were considered to be novel. Note that, as described in [11], Ensembl transcript models were generated from a combination of previously described *Anopheles *protein sequences, high homology matches from SwissProt+TrEMBL and *Anopheles *EST information. In our analysis, we do not consider transcript models generated only from *in silico *gene-prediction algorithms. In summary, when we find a cluster that does not overlap any predicted Ensembl transcript, the cluster is considered the product of a new gene and is designated as being 'novel'. If a cluster does overlap an Ensembl transcript, it is classified as known even if the initial evidence for that gene relies, for example, on homology alone.

Using this approach we find that 3,032 clusters (86%) correspond to predicted Ensembl genes. Of these, nearly 46% (1,393 sequences) extend both the 5' and 3' ends of Ensembl-predicted transcripts. In addition, 9% extend the 5' end only (271 clusters) and 31% extend only the 3' end (935 clusters) of the corresponding Ensembl transcript. Just 433 clusters (around 14%) fell entirely within a predicted gene and did not extend either extremity of an Ensembl gene model. In addition, 536 clusters that do not correspond to any previously described Ensembl gene were also identified. The mean length of these novel clusters was 1,303 nucleotides versus 1,615 for Ensembl-predicted genes. As detailed in Table 1, both Ensembl-predicted and novel clusters appear to be well distributed across the genome. As expected, the majority of clusters are composed of a small number of reads - 37% of clusters have two to three reads and 80% contain fewer than 12. The single cluster with the greatest number of reads (over 2,000) is annotated as a guanine-nucleotide-binding beta subunit.

While consistent, this method does require that some qualifying conditions be kept in mind. First, it is possible that a gene that we designate as novel does in fact have some previous transcript information available as supporting evidence (such as EST data). This will happen, for example, if during the process of automatic annotation the existing information did not result in the creation of a new transcript model by Ensembl. In fact, in the initial analysis of the *Anopheles *genome, as many as 1,029 genes were believed to have been missed in this manner [2]. Since the initial annotation process, the increased amount of available sequence information has improved coverage considerably. Despite these improvements, however, such misclassifications are unavoidable. In addition, if an Ensembl prediction is incorrect, an overlapping cluster would be classified as previously predicted, although it would, in fact, be new to the annotation. Inspection shows that such instances are rare and generally require additional experimental evidence as well as the manual definition of gene models for complete reconciliation of the data. While difficulties will exist with any such automated comparison, as a whole our approach is consistent, reproducible, and provides realistic estimates of both previously predicted and novel genes.

Of the initial set of 10,961 singlets (see Figure 1), most (around 80%) not only aligned to the genome with high quality, but also overlapped with Ensembl predictions, while approximately 2,200 singlets were unable to be aligned. This latter group is discussed further in the next section. Additionally, 202 reads or 'singlets' were found that accurately aligned to the genome but did not overlap with any Ensembl transcript predictions. These singlets are generally shorter in length than clusters, with a mean of 912 nucleotides. Of the 202 sequences, 65 were found through manual examination to be probable 5' or 3' extensions of a nearby Ensembl-predicted transcript. Of the remaining 137 singlets, 38 (or 28%) are non-overlapping 5- and 3-prime reads representing 19 genes where additional sequencing must be done to obtain the complete gene sequence. Blastx analysis against a combined SwissProt+TrEMBL database showed that 25 of the novel singlets (around 12%) have limited homology to previously described genes (E-value < 10^-7^), with the remaining novel singlets having no significant similarity to the database members. Thus singlets provide evidence for 118 additional novel genes, and together with the previously described clusters, support the existence of 654 novel genes. While clusters supported by a singlet provide further opportunities to investigate potentially novel genes, we do not describe them further here. Future work will investigate such transcripts in greater detail.

### Unalignable sequences

We note that 2205 sequences (around 3% of all reads) cannot be aligned to the genome. Essentially all of these sequences are singlets, many of which are of low complexity and/or contain repetitive regions. Nearly half (1,066) were eliminated during the alignment process due to their poor quality (identity and/or coverage). It is possible, however, that some unaligned sequences represent genes lying within sequence gaps of the genome assembly. For example, within the unalignable group there are eight clusters having an average length of 1250 nucleotides, composed of from two to four reads, with three of these clusters consisting of overlapping 5' and 3' reads. Visual inspection suggests that most of these clusters also contain low-complexity regions. In addition, in two cases Blastx [12] searches against a nonredundant protein database reveal similarity to known proteins. One cluster has high similarity to a receptor for activated protein kinase C (RACK1; E-value ~10^-62^) while the second has similarity to a putative ribosomal protein (S8; E-value ~10^-12^). Comparing the remaining 1,139 reads that could not be initially aligned to any chromosome arm via BLAST, we found that at least 808 reads appear to be bacterial contaminants. Approximately 19% have no similarity to proteins in SwissProt+TrEMBL. Another 10% of the group (118 sequences) have similarity to known proteins (E-value < 10^-7^). In fact, 32 sequences have similarity to previously identified *Anopheles *proteins. At this time, it is not clear whether these sequences fall into unsequenced gaps in the genome sequence, are of insufficient quality to align accurately, or are errors or some other artifact. While it is possible that many of these sequences that could encode proteins with similarity to known proteins are actual gene transcripts, we do not consider them further here and do not include them in our group of novel genes.

### Characterization of Ensembl-predicted and novel cDNA clusters

**Figure 2:**
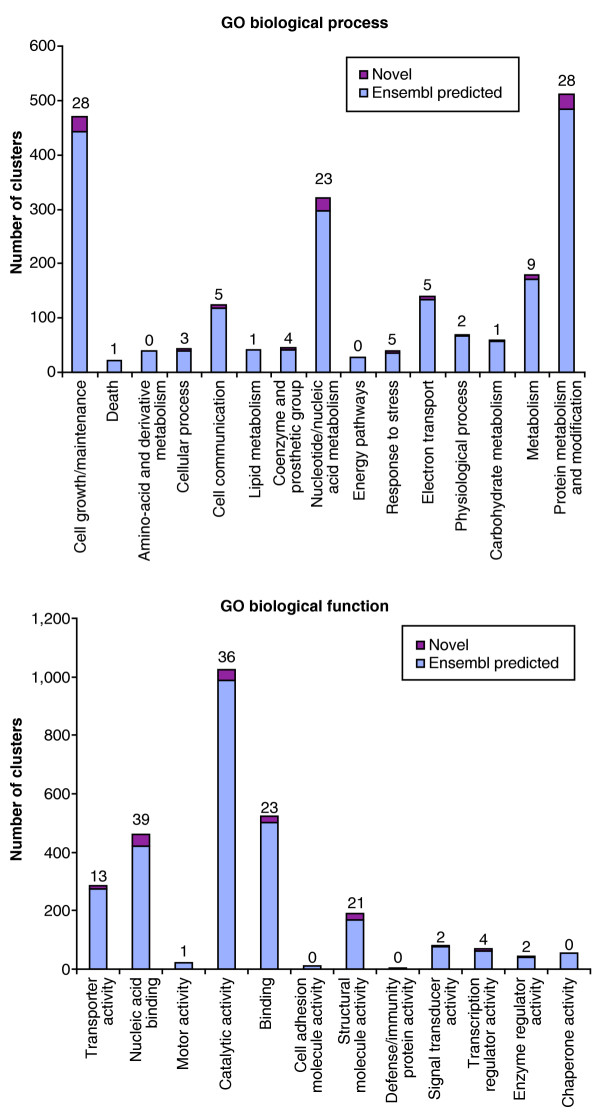
Classification of clusters with Gene Ontology. Numbers above bars indicate the number of novel clusters in the given category.

To characterize cDNA clusters in terms of their potential biological role, we compared both Ensembl-predicted as well as novel gene clusters to a Gene Ontology (GO) annotated database (SwissProt+TrEMBL 796,016 sequences) [13,14]. Using Blastx and an E-value of 10^-7^, clusters could be placed into a range of biological processes and functions (Figure 2). For the clusters supporting Ensembl-predicted genes, 2,398 of 3,032 (79%) could be assigned to a biological process or function, as compared to the novel clusters where only 123 out of 536 (23%) had at least one qualifying match. Of the deduced proteins of clusters corresponding to predicted genes, approximately 63% could be classified as having catalytic, binding, or nucleic-acid-binding function. Similarly, for deduced proteins of novel gene clusters, these same categories were the most highly populated, representing nearly 80% of classified functions. The processes of cell growth and/or maintenance and protein metabolism and modification were the most highly represented process categories for both Ensembl-predicted and novel cDNA clusters.

To better describe the novel genes defined by the cDNAs, we compared consensus sequences from each cDNA cluster to a SwissProt+TrEMBL database and found that approximately 35% (188) of novel clusters had significant hits to known proteins (E-value 10^-7^). Again, these clusters were represented by a single consensus sequence composed of between two and 19 reads. For those transcripts without significant homology results, it is likely that many represent species-specific and/or insect-specific genes, and are thus of particular interest for more detailed experimental study.

In addition, we attempted to identify a satisfactory open reading frame (ORF) in each cluster. Of the 536 novel clusters in the final set, 298 contained an ORF of at least 100 amino acids (see Materials and methods). Additional evidence in support of the biological reality of a gene or gene transcript is the existence of protein domains within the ORF as well as multi-exonic structure. As shown in Table 2, we found 60 ORFs encoding at least one Pfam domain. Most domains are found only once; the zinc finger C2H2 domain is found 18 times, however, distributed over five different proteins. Further evidence in support of these clusters being real biological entities is the observation that nearly half of the clusters (47%) are comprised of two or more exons.

### GC content of cDNA clusters

**Figure 3:**
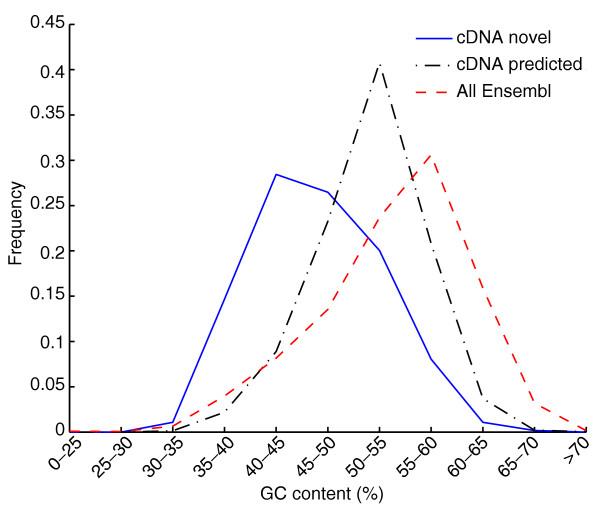
GC content of cDNA clusters and Ensembl transcripts.

It has been suggested that, at least in the case of human genome annotation, there is a prediction bias against GC-rich transcripts by current gene-prediction methods [15]. To investigate the possibility that there are obvious biases in sequence properties of novel clusters that would make them more or less difficult to predict computationally, we determined the GC content for novel and predicted cDNA clusters and compared them to all Ensembl-predicted genes. As shown in Figure 3, the Ensembl transcript models are largely contained between 35 and 70% GC content with a mean of 54%. The range of GC content for both novel and predicted clusters spans a nearly equivalent range. For the novel clusters, however, there appears to be bias towards more AT-rich transcripts. The mean GC content for novel clusters was 46%, compared to 52% for clusters corresponding to predicted genes. As a whole, the *Anopheles *genome has a GC content of 35.2% (*Drosophila melanogaster *is 41.1%) [2]. As a simple test, we compared novel clusters to geneid [16] predictions and found that 232 clusters (43%) overlap with a geneid prediction, while 311 novel clusters (57%) do not. In contrast, only 9% of Ensembl-predicted genes do not have a corresponding geneid prediction. This result suggests that the majority of novel genes would not be readily discovered without customized gene-finding methods. Currently, newer gene-finding methods specifically trained on *Anopheles *cDNAs are now being developed and implemented (see, for example [17]) into the Ensembl gene prediction and annotation methodology (E. Mongin, personal communication).

### Examples of Ensembl-predicted and non-predicted clusters

As discussed earlier, genes represented by full-length cDNA transcripts span a wide range of molecular and cellular roles. Here we highlight a few examples and their relevance to current *Anopheles *research. Note that we have compared these transcripts to a more recent version of the Ensembl database (release 23) that now includes these cDNAs as part of the gene build process. As a result, our cDNA transcripts are identified in this section by their current ENSANGT, ENSANGEST, or name identifier whenever appropriate. While some of the genes described here had previous EST evidence, the availability of full-length enriched cDNAs for these transcripts is particularly valuable for future annotation.

**Figure 4:**
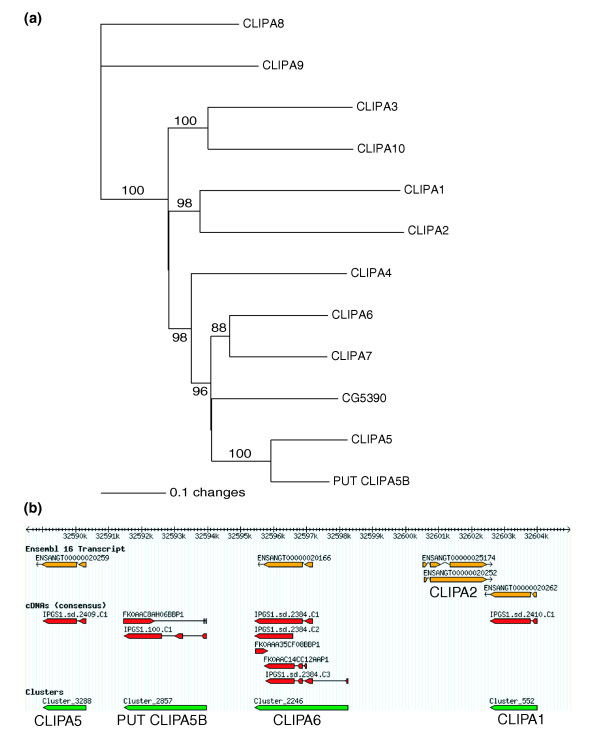
Putative novel member of the CLIPA protein subfamily. **(a) **Phylogenetic tree of CLIPA subfamily proteins and the novel member described here - PUT CLIPA5B. The protein CG5390 is the closest *Drosophila *relative to this protein. Bootstrap values are shown as percentages of 1,000 replications (see Materials and methods). **(b) **Genomic region containing the putative gene. Yellow bars indicate Ensembl 16 transcripts, cDNA evidence is shown in red and cDNA clusters are shown in green. Similarity and proximity suggest that this novel member probably arose through a recent duplication of CLIPA5.

One transcript of interest encodes a protein containing both CLIP and serine protease domains. This protein, which we have designated here as Putative_CLIPA5B, has been incorporated into Ensembl as part of transcript ENSANGT00000027174. In insects, these CLIP-domain serine proteases are involved in a variety of processes, including embryonic development and the innate immune response. For example, in response to malarial infection, CLIP-domain proteases help to initiate the prophenoloxidase cascade which, in 'malaria-resistant' mosquitoes, results in the generation of reactive oxygen species and the eventual encapsulation of the parasite within a melanin capsule [18,19]. Four subfamilies (A-D) are known within *Anopheles*, and phylogenetic analysis of the novel protein sequence deduced from our novel cluster suggests that it is a new member of the A subfamily (Figure 4a). Ten members of this family were previously described and CLIPA5 appears to be the closest relative of the new protein. The gene for the new protein lies within a cluster of 15 serine protease/CLIP-domain genes located on chromosome arm 3L (between 32.55-32.62 MB). Its similarity and proximity to *clipA5 *would suggest that it arose from a recent duplication event. While the exact function of this new protein is unknown, it is interesting to note that transcription of a related member in the same subfamily, *clipA6*, is induced by bacterial infection [20].

**Figure 5:**
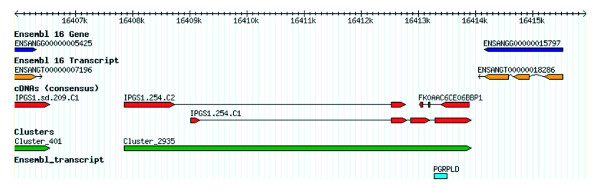
Peptidoglycan recognition protein LD. Cluster of cDNAs (cluster_2935 in green) associated with the peptidoglycan recognition protein LD. Note that the current Ensembl definition of PGRPLD (in cyan) is truncated and does not currently reflect available transcript information.

We also identify a cDNA that encodes a peptidoglycan recognition protein (gene D of the long (L) subfamily - PGRPLD). Members of this protein family play a key role in the response to both bacterial and malarial infection [21]. While PGRPLD was not predicted in the original *Anopheles *annotation and was not part of the Ensembl 16 annotation, it was predicted without cDNA evidence in the preliminary analysis of immune genes within the genome [22] (Figure 5). In *Drosophila*, PGRPLD is enriched in hemocytes, is probably membrane bound and is actively expressed throughout development. Although its exact role in innate immunity is currently unknown, it is believed to be involved in bacterial recognition [23]. As many as three different gene products may be produced by *pgprld *in *Drosophila*, and our full-length cDNAs suggest two alternative start sites for this gene in *Anopheles*. Interestingly, as described in *Drosophila*, its untranslated 3' end overlaps with an ORF on the opposite strand encoding retinaldehyde-binding and alpha-tocopherol transport domains [23]. The cDNAs for *pgprld *have been incorporated into the supporting evidence for Ensembl EST transcript models ENSANGESTT00000363407 and ENSANGESTT00000363376.

Other transcripts of interest are two previously non-predicted, putative P450 genes, which are of particular interest with regard to insecticide resistance. Currently, the major method for mosquito control within malaria endemic regions is the use of pyrethroid-based insecticides, typically through the impregnation of bednets and application to mosquito breeding sites [24]. While a major tool in the fight against malaria, the continued development of mosquito resistance to these insecticides has become an important problem. One potential mechanism of resistance to insecticides is the oxidative metabolism of insecticides mediated by cytochrome P450 [25,26]. While definitive proof of the involvement of P450 in resistance is limited [27], it has been shown that certain P450 families are expressed at higher levels in various insecticide-resistant strains (see, for example [28,29]). Of the two putative P450 genes discussed here, one (ENSANGT00000029062) has high similarity (E = 10^-146^) to CYP9L1 and the other has similarity to CYP6M4 (E = 10^-149^; Ensembl known transcript AAP76391). Both families are insect-specific, and members of the *Cyp6 *family have been linked to insecticide resistance by elevated P450 activity in insecticide-resistant insects [25]. In total, we retrieved cDNAs representing 23 of the known 111 members of the *Anopheles *P450 family.

We also find examples of interesting novel genes that are currently found only within this cDNA library. For example, our cDNAs identify a 869-base-pair (bp) gene (ENSANGT00000025538) which is most similar to mouse and human members of the MAGE (melanoma antigen-encoding) gene family. This gene was previously unrecognized in *A. gambiae *even though a *Drosophila *member of this family does exist [30]. The gene was previously found to be expressed specifically in mammalian tumors and is developmentally regulated in *Drosophila *[30]. Another example is a transcript of approximately 1,300 bp which is homologous to *Drosophila *DIP2 (Disco interacting protein 2, CG9771) which is involved in nervous system development [31].

## Conclusion

We found that over 85% of previously predicted *A. gambiae *genes had their boundaries extended either on the 5', 3', or both 5' and 3' ends by our initial full-length cDNA collection. While all the consensus models are not complete full-length transcripts, it is particularly encouraging that such a large percentage of previously predicted gene models were extended on both their 5' and 3' ends. The use of such full-length data is particularly valuable in the absence of well annotated and evolutionarily close organisms which can be used for sequence comparisons. The sequencing of the *Aedes aegypti *genome is much anticipated in this regard. However, even with the availability of a genome from a mosquito relative, species-specific genes, along with the variability inherent in 5' and 3' exons, will probably require the use of full-length data for accurate gene characterization.

A major result of this study was the finding that approximately 17% of the clusters represent previously unpredicted genes. This is perhaps more significant when considering that this was a non-normalized library constructed from whole mosquitoes. Further extrapolation suggests that at least a similar percentage of genes remains to be found elsewhere in the *Anopheles *genome. Additional tissue- and treatment-specific libraries, currently under construction, should help to characterize more undiscovered genes.

Note added in proof: A recent status report of the *Anopheles *annotation effort by Ensembl agrees with our estimates suggesting that around 600 new genes were discovered from the sequences presented in this communication, and that the current transcript set may be under-represented by as much as 20% [11].

## Materials and methods

### Construction of oligo-capped cDNA libraries

Total RNA (cytoplasmic RNA and poly(A)^+ ^RNA) was isolated from 1,366 adult female *A. gambiae *strain 6-9 mosquitoes, collected 24 h after oviposition by homogenization of the insects in TriReagent (Sigma) with an Ultra-Turax T25 homogenizer (IKA-Werke, Germany) as recommended by the suppliers.

The isolated total RNA was resuspended in H20 and the poly(A)^+ ^RNA fraction was obtained from the equivalent of 700 μg total RNA using the Qiagen Oligotex mRNA batch protocol. Oligo-capped libraries were then constructed from the poly(A)^+ ^RNA fraction as described by Sugano and collaborators [32,33]. Synthesis of the first-strand cDNA was obtained with the SuperScriptII RNase H-Reverse Transcriptase (Invitrogen); subsequently, the template RNA strand was degraded by alkaline hydrolysis and the first-strand cDNA was amplified using the LA Taq polymerase (Takara). After 20 PCR cycles the PCR fragments were digested with *Sfi*I and size-fractionated by agarose gel electrophoresis. Two different size fractions (0.7-1 kilobase (kb), 1 kb-3 kb) were cloned into the vector pME18S-FL3 in an orientation-defined manner, using a DNA ligation kit (Takara). Ligations were electroporated into *Escherichia coli *DH10B electrocompetent bacteria (Invitrogen). Clones were randomly isolated and subjected to high-throughput single-path sequencing from their 5' and 3' ends. Note that this is a female whole-body library created under the constraints of selection for full-length transcripts within a given size range, and as such, does not provide a comprehensive survey of the genes expressed or capable of being expressed within the female *Anopheles *mosquito.

### Availability of libraries

All libraries/clones are being deposited to MR4 and will be available there [10].

### Sequence clustering, assembly and comparison

Sequences were cleaned, clustered and assembled using the Paracel TranscriptAssembler software package (Paracel). Cleaning consisted of comparing cDNA sequences against vector and mitochondrial databases, with matching sequences being removed from further analysis. In addition, low-complexity, poly(A/T) regions, and repeat regions (Ensembl repeat library courtesy of E. Mongin, Ensembl) were determined and masked. After sequence cleaning, masking and trimming, sequences with fewer than 200 unmasked bases were removed from further processing. As an aid to the initial clustering process, we used Ensembl release 16 cDNA transcripts as seed clusters. In this process, each cDNA is compared to each Ensembl transcript, and if significant similarity exists between the two, the cDNA is placed into a corresponding seed bin and clustered with all transcripts in this bin. Sequences that did not have high similarity to seed sequences were separately compared and clustered with each other. Next, both seed and non-seed clusters were assembled into one or more consensus sequences. If a sequence could not be assembled into the consensus sequences it was designated as a singlet. Finally, each consensus and singlet sequence was aligned to the Ensembl *Anopheles *genome assembly (release 16.2.1) using a combination of BLAST and Spidey [12,34] with minimum identity and coverage of 90% and 75% respectively. In addition, to prevent spurious 'exons' from being produced from low-quality sequence noise common at read extremities, we trimmed terminal exons separated by over 10 kb and which were less than 50 nucleotides long.

We compare the resulting clusters and singlets to Ensembl transcripts (ENSANGT identifiers) from the *Anopheles *16.2.1 release. Note that database revision numbers between 16.2.1 and 20 contain only one new gene build (ver. 17.2a.1 which incorporates the cDNA sequence data presented in this paper) with the rest primarily representing changes to the underlying database schema. If a cluster did not overlap on the genome with an Ensembl gene, it was classified as 'novel'; otherwise it was classified as 'Ensembl predicted'. The protein database used for homology searches was a combined Swiss-Prot (Release 44.2) and TrEMBL (Release 27.2) dataset.

Internally, we used the Genome Browser (Gbrowse) [35] developed by the Generic Model Organism Database consortium [36] for display and analysis of clusters as well as the public resources provided by Ensembl [3].

### Gene Ontology terms

We used the following terms and GO IDs in the creation of Figure 2:

Biological Process-cellular process; GO:0009987, cell communication; GO:0007154, physiological process; GO:0007582, metabolism; GO:0008152, carbohydrate metabolism; GO:0005975, energy pathways; GO:0006091, electron transport; GO:0006118, nucleotide and nucleic acid metabolism; GO:0006139, amino-acid and derivative metabolism; GO:0006519, protein metabolism and modification; GO:0006411, lipid metabolism; GO:0006629, coenzymes and prosthetic group metabolism; GO:0006731, cell growth and/or maintenance; GO:0008151, death; GO:0016265, response to stress; GO:0006950.

Biological Function-cell adhesion molecule activity; GO:0005194, chaperone activity; GO:0003754, GO:0003757, GO:0003758, GO:0003760, GO:0003761, defense/immunity protein activity; GO:0003793, catalytic activity; GO:0003824, enzyme regulator activity; GO:0030234, binding; GO:0005488, nucleic acid binding; GO:0003676, motor activity; GO:0003774, signal transducer activity; GO:0004871, structural molecule activity; GO:0005198, transcription regulator activity; GO:0030528, transporter activity; GO:0005215.

### CLIPA phylogenetic tree

The regions containing the CLIP and serine protease domains for each sequences were aligned with ClustalX [37] (default values; version 1.83), manually adjusted in Jalview, and a neighbor-joining tree created, excluding gaps, with PAUP*. The CLIP and serine protease domains were included in the alignment and large insertions were removed before aligning.

### ORF determination

For each cluster considered, a representative cDNA sequence was taken (the longest in terms of total concatenated exon length if there were multiple consensus sequences in a cluster) and translated in all six reading frames. An ORF was defined as being at least 100 codons long, starting with a methionine and ending with a stop codon.

## Figures and Tables

**Table 1:** Distribution of cDNA clusters across the *Anopheles *genome

	2R	2L	3R	3L	X	UNKN
Predicted	950 (31%)	676 (22%)	588 (19%)	439 (15%)	229 (8%)	146 (5%)
Novel	130 (24%)	141 (26%)	99 (18%)	92 (17%)	50 (9%)	31 (6%)

**Table 2:** Pfam domains within novel ORFs

Pfam domain	Description	Number
adh_short	Short chain dehydrogenase	1
Aldo_ket_red	Aldo/keto reductase family	1
Amidase_2	*N*-acetylmuramoyl-L-alanine amidase	1
Ank	Ankyrin repeat	3
Bin3	Bicoid-interacting protein 3 (Bin3)	1
CBFD_NFYB_HMF	Histone-like transcription factor (CBF/NF-Y) and archaeal histone	1
CH	Calponin homology (CH) domain	1
CRAL_TRIO	CRAL/TRIO domain	1
Death	Death domain	1
DEP	Domain found in Dishevelled, Egl-10, and Pleckstrin	1
Dsrm	Double-stranded RNA binding motif	2
DUF1395	Protein of unknown function (DUF1395)	1
DUF227	Domain of unknown function (DUF227)	1
DUF783	Protein of unknown function (DUF783)	1
Efhand	EF hand	4
Exonuc_X-T	Exonuclease	1
F-box	F-box domain	1
FYRC	F/Y rich C-terminus	1
G_glu_transpept	Gamma-glutamyltranspeptidase	1
GST_C	Glutathione S-transferase, C-terminal domain	1
HIT	HIT domain	1
Ins_allergen_rp	Insect allergen related repeat	1
Linker_histone	Linker histone H1 and H5 family	1
LRR	Leucine rich repeat	4
LSM	LSM domain	1
MtN3_slv	MtN3/saliva family	2
p450	Cytochrome P450	2
Pkinase	Protein kinase domain	1
Psf2	Partner of SLD five, PSF2	1
Radical_SAM	Radical SAM superfamily	1
Retrotrans_gag	Retrotransposon gag protein	1
Ribosomal_L27e	Ribosomal L27e protein family	1
Ribosomal_L36e	Ribosomal protein L36e	1
Ribosomal_L37e	Ribosomal protein L37e	1
Ribosomal_S8	Ribosomal protein S8	1
RRM_1	RNA recognition motif. (a.k.a. RRM, RBD, or RNP domain)	1
SAM_1	SAM domain (sterile alpha motif)	1
Serpin	Serpin (serine protease inhibitor)	1
Tetraspannin	Tetraspanin family	2
THAP	THAP domain	2
TIL	Trypsin inhibitor like cysteine rich domain	1
TIP49	TIP49 C-terminus	1
TPR	TPR Domain	1
TraB	TraB family	1
Trypsin	Trypsin	1
Tubulin	Tubulin/FtsZ family, GTPase domain	1
Tubulin_C	Tubulin/FtsZ family, C-terminal domain	1
UNC-50	UNC-50 family	1
UPF0224	Uncharacterized protein family (UPF0224)	1
WD40	WD domain, G-beta repeat	3
zf-C2H2	Zinc finger, C2H2 type	18
zf-C3HC4	Zinc finger, C3HC4 type (RING finger)	1
